# Traumatic fractures of the sternum – typical distribution and need for subgroups within AO and OTA classification system?

**DOI:** 10.1007/s00068-025-02910-x

**Published:** 2025-06-27

**Authors:** Johannes Groh, Florian Kern, Anne Schenderlein, Johannes Krause, Mario Perl, Stefan Schulz-Drost

**Affiliations:** 1 Department of Trauma and Orthopedic Surgery, University Hospital Erlangen Friedrich-Alexander University Erlangen-Nürnberg (FAU), Erlangen, Germany; 2 Faculty of Medicine, Department of Anesthesiology, Friedrich-Alexander-Universität Erlangen-Nürnberg, Erlangen, Germany; 3 Department for Trauma Surgery, Helios Kliniken Schwerin, Schwerin, Germany

**Keywords:** Sternum fracture, Sternal fracture, Chest wall injury, Manubrium, AO/OTA classification system

## Abstract

**Introduction:**

Objective of this study is the morphologic validation of the AO-classification of the sternal bone and particularly define subgroups.

**Methods:**

Analyzed were all patients of a level I trauma center of a 7-year period with fractures of the sternal bone and the anterior rib cartilage. A total number of *n* = 124 patients was included. The detailed evaluation of the CT-data recorded anatomical basic data of the rib cage and every fracture with its position, dislocation, fracture pattern (which was classified following the AO).

**Results:**

116 (93.5%) patients showed 134 single fractures of the sternal bone, 48 (35.8%) of the manubrium, 81 (60.4%) of the corpus sterni and 5 (3.7%) of the xyphoid. 16 patients had a dual fracture of manubrium and corpus. Fractures of the corpus were mostly type A-fractures, followed by type B-and C-fractures. Manubrial fractures had the same number of type A- and B-fractures. Subgroups were theoretically defined by the senior author of our group and validated. based on location, dislocation and course of the fracture.

**Discussion:**

Sternal fractures are mostly shown at the corpus. Fractures of the xiphoid are very uncommon. Generally, corpus fractures are simple fractures, the rarer manubrium fractures show more complex fractures, which presumes a high trauma energy. The defined subgroups can help draw conclusion to the trauma mechanism and its potential concomitant injuries.

## Introduction

After traumatic brain injury, thoracic trauma is classified as the second most common cause of accidental death. This quickly becomes understandable when one considers the anatomical complexity of the thorax with its vital organs, vessels and the surrounding bones. The biomechanical structure of the thorax plays an essential role in ensuring respiratory physiology and at the same time performing protective functions for the thoracic organs.

The interaction between the trunk statics, the dorsal spine with its twelve thoracic vertebral bodies, and the corresponding twelve pairs of ribs as well as the sternum as the anterior keystone of the chest wall is of outstanding importance. In addition, the anterior shoulder girdle attaches to the sternum and the clavicle joint on both sides and forms the only bony connection to the trunk, which is the basis for the function and mobility of the arms.

It should be mentioned that the anterior chest wall also plays an important role in the trunk statics and has been included in the stabilizing effect of the sagittal profile for many years, even being considered the fourth column of the stability of the thoracic spine [1, 2].

Injuries to the anterior chest wall can have significant consequences with a vital threat as well as functional limitations. In recent years, inpatient fractures have become increasingly important. By 2019, there had been an increase of 40% in inpatient rib series fractures and around 15% in sternal fracture [3]. This trend is clearly increasing, as more older people in particular are suffering from these injuries and diagnostics have also been intensified and their quality has been significantly improved over the years [4].

By far not all fractures are visible in the normal X-ray image; therefore, CT diagnostics has established itself as the standard for identifying fractures and describing them morphologically. In the context of acute diagnostics, but also in the follow-up examination of chronic complaints, ultrasound and magnetic resonance imaging are also gaining in importance [5, 6].

Although rib and rib series fractures are treated much more frequently than sternal fractures, the sternum plays a central role. Overall, sternal fractures are relatively rare compared to other bone fractures. In severely injured patients, sternal fractures occur in about 3.9% of cases, while almost half of all severely injured patients suffer rib fractures. Together with rib fractures, they account for eight to over 20%, depending on the severity of the injury. In combination with rib fractures, sternal fractures contribute significantly to increased mobility and mortality of severely injured patients [7].

The development of sternal fractures can occur through both direct and indirect force on the trunk [8, 9]. Direct force can be caused, for example, by crushing the chest or punctual forces on the sternum. Examples of this are the impact of vehicle occupants against the steering wheel, acts of violence with blows to the front chest wall or resuscitation attempts. More extensive compression of the thorax can also occur if a seat belt is worn during an accident or if the body is trapped.

Indirect force can break the sternum through various mechanisms such as flexion or compression, more rarely through hyperextension or distraction mechanisms [10]. The anterior shoulder girdle also plays a role in sternal fractures, especially at the manubrium. Oblique manubrium fractures were associated with a high attachment of the seat belt in the vehicle [11]. In flexion and compression injuries, a fracture can occur due to the impact of the chin on the manubrium [12].

This large number of injury mechanisms, the three bone parts of the STERNUM with the manubrium corpus and xiphoid as well as the interaction with adjacent ribs, the spine and possible thoracic organ involvement makes it difficult to compare fractures with each other.

Numerous research groups have therefore investigated the possible treatment options for sternal fractures. The focus is on the question of whether surgical therapy, for example by plate osteosynthesis, offers advantages over conservative therapy. While the technical implementation was safe and successful in the vast majority of cases, various results on the clinical outcome have been published. Some studies show that improved survival rates were found with surgical therapy, while the inpatient length of stay after surgery was longer [13]. Case-control studies with differentiated analyses show better clinical outcomes in some cases, especially in relation to pain and upper extremity mobility. However, studies on large patient databases have not been able to consistently prove this superiority [14–16]. None of the studies used morphological criteria of the fractures to compare the therapeutic successes.

In order to make sternal fractures more comparable and to evaluate their clinical relevance, a classification system was required. This was first published by the AO (Working Group for Osteosynthesis Issues) and OTA (Orthopedic Trauma Association) in 2018 and contains basic morphological descriptions of the fractures of the manubrium, the corpus sterni and the xiphoid process [17]. Inter-Observer analysis has shown that classification is generally relatively feasible, but some important aspects still need to be revised. For example, the course of the fracture should be described in more detail and presented in two planes– coronary and sagittal [18]. The transitions of the bones must also be named more precisely. So far, no subtyping has been named.

The present study focuses on investigating sternal fractures with regard to their morphology, frequency and the occurrence of possible subgroups. The primary endpoints are the frequency of fractures and the distribution by gender and age. In addition, the occurrence of the main injury mechanisms is classified according to ABC (A– B– C). The secondary endpoints focus on possible subtypes of fractures, concomitant injuries and accident mechanisms.

## Materials and methods

A retrospective analysis had been carried out. All inpatients of one level one trauma center had been included if they suffered traumatic rib or sternum fractures during the years 2010–2016. The total of 1734 patients were first analyzed for CT scan, all patients without CT scan (914) were excluded. The remaining 820 patients and their CT scans were also checked for suitability for the study. A further 126 patients were excluded if they had received a CT scan, but this was either not temporally related to the fracture event, had inadequate image quality (e.g. insufficient slice thickness, only rudimentary imaging of the thoracic wall, etc.) or had iatrogenic fractures caused by surgery (e.g. osteotomies) on the thorax or already consolidated fractures.

This left 694 patients for further analysis. After removal of duplicates, a collective of 632 patients remained to be analyzed.

An overview of the inclusion and exclusion criteria can be found in Table 1.

**Table 1:** Inclusion and exclusion criteria

Inclusion criteria	Exclusion criteria
Inpatient treatment	Iatrogenic fractures
Fracture of the ribs and/or sternum	Fractures missing on CT despite coding
Presence of usable CT diagnostics of the thoracic wall with fractures	Consolidated fractures
Defined period	Non-usable imaging

The remaining 632 patients were divided into three sub-collectives (anterior sub-collective, lateral sub-collective and posterior sub-collective) by analyzing the CT findings and screening the CT images.

The subcollective analyzed in this study contains all patients who had at least one fracture of the anterior chest wall. The anterior chest wall was defined here as a fracture of the sternum or the costal cartilage including the osteochondral junction. This left 142 patients.

In the course of the evaluation, another 18 of the original 142 patients had to be excluded. The CT scans of six patients could not be opened for unexplained technical reasons, ten patients showed either no fracture or a fracture outside the anterior sector despite the previously analyzed radiological findings. Two patients were removed due to duplication and were assigned a new case number during their inpatient stay.

This resulted in a total collective of *n* = 124 patients to be analyzed. 116 of these Patients showed fractures of the sternal bone, this group is discussed in this paper.

**Fig. 1:**
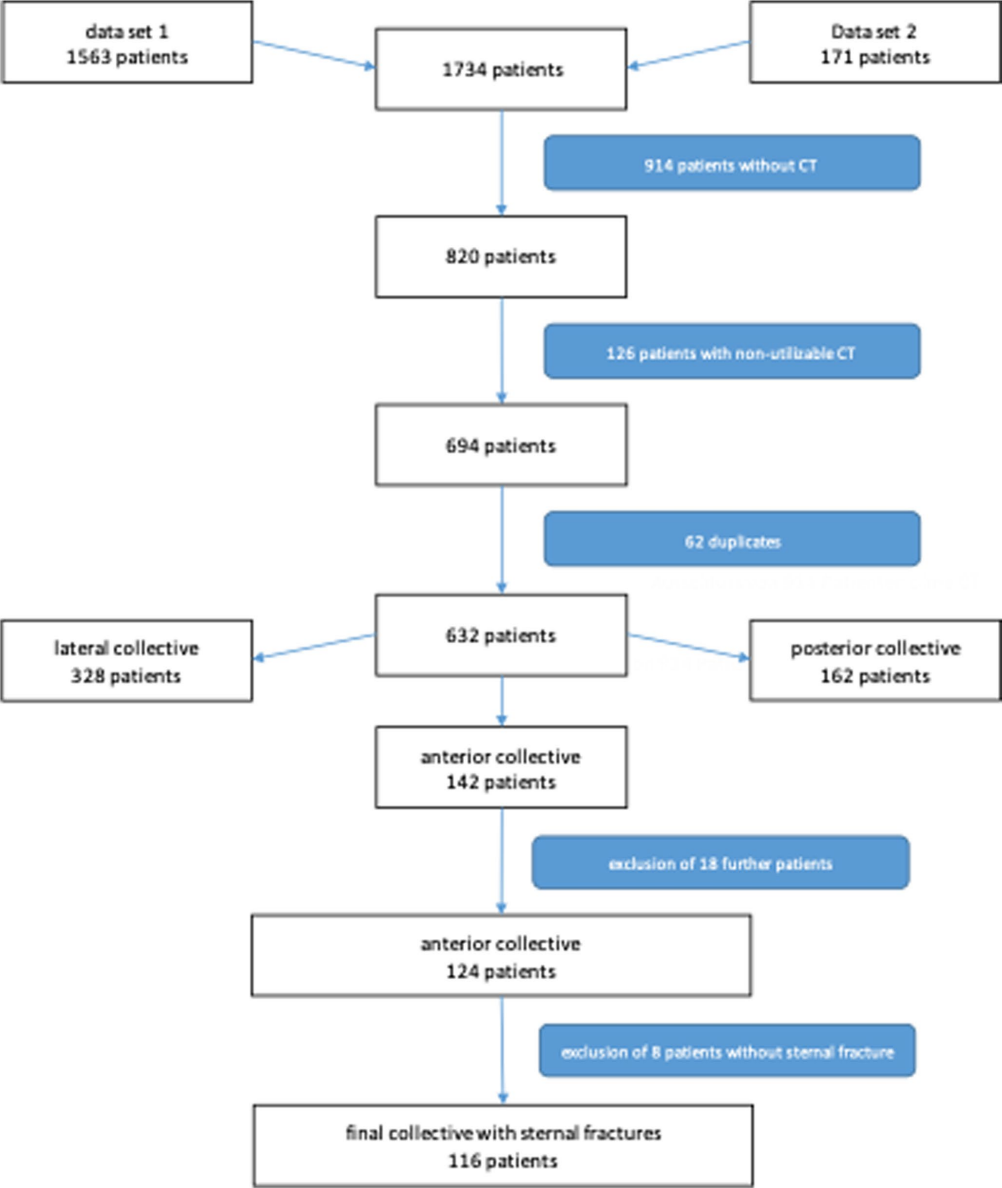
Creation of the patient collective

The following flowchart provides an overview of the process of creating the collective (Fig. 1).

First, basic anatomical data of the bony hemithorax and epidemiological data of the collective were analyzed. All fractures of the anterior chest wall, in this case the sternal fractures, were classified according to their location, dislocation and fracture type according to the AO/OTA classification system. Possible subtypes were also analyzed.

If a fracture of the respective structure was present, it was measured precisely.

**Fig. 2:**
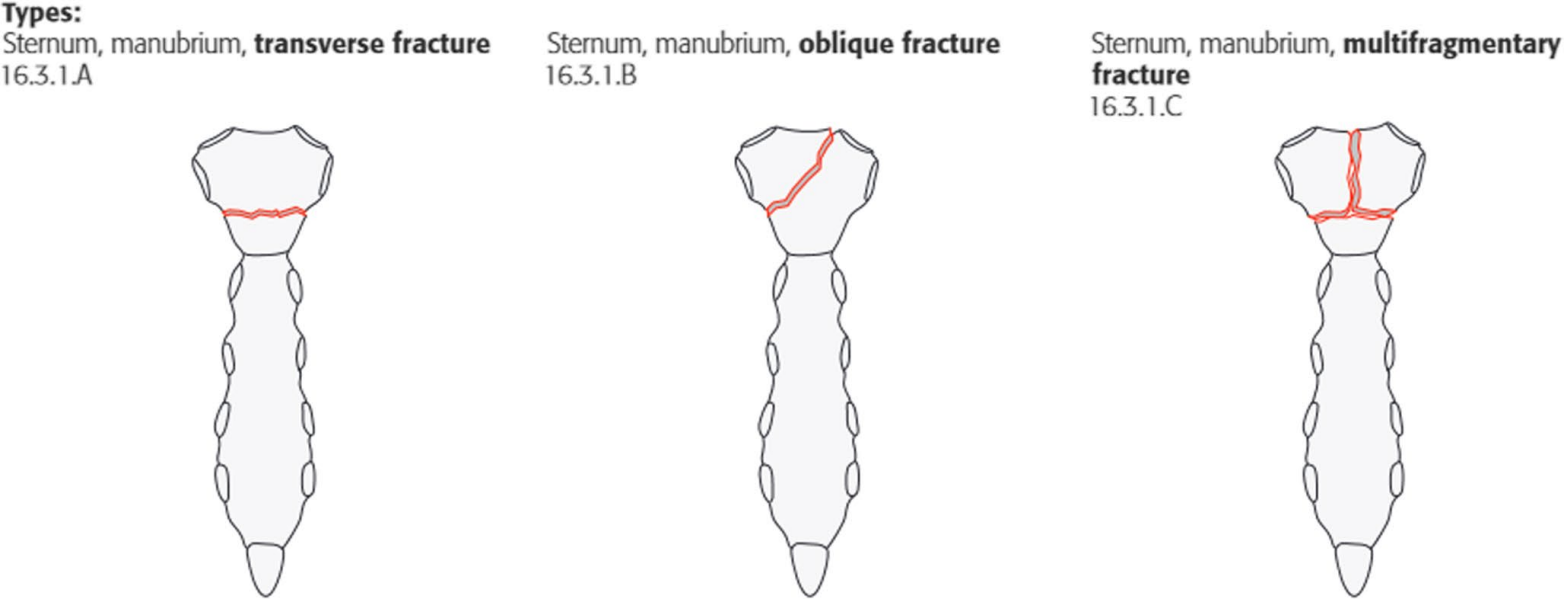
Fracture types at the manubrium [17]

**Fig. 3:**
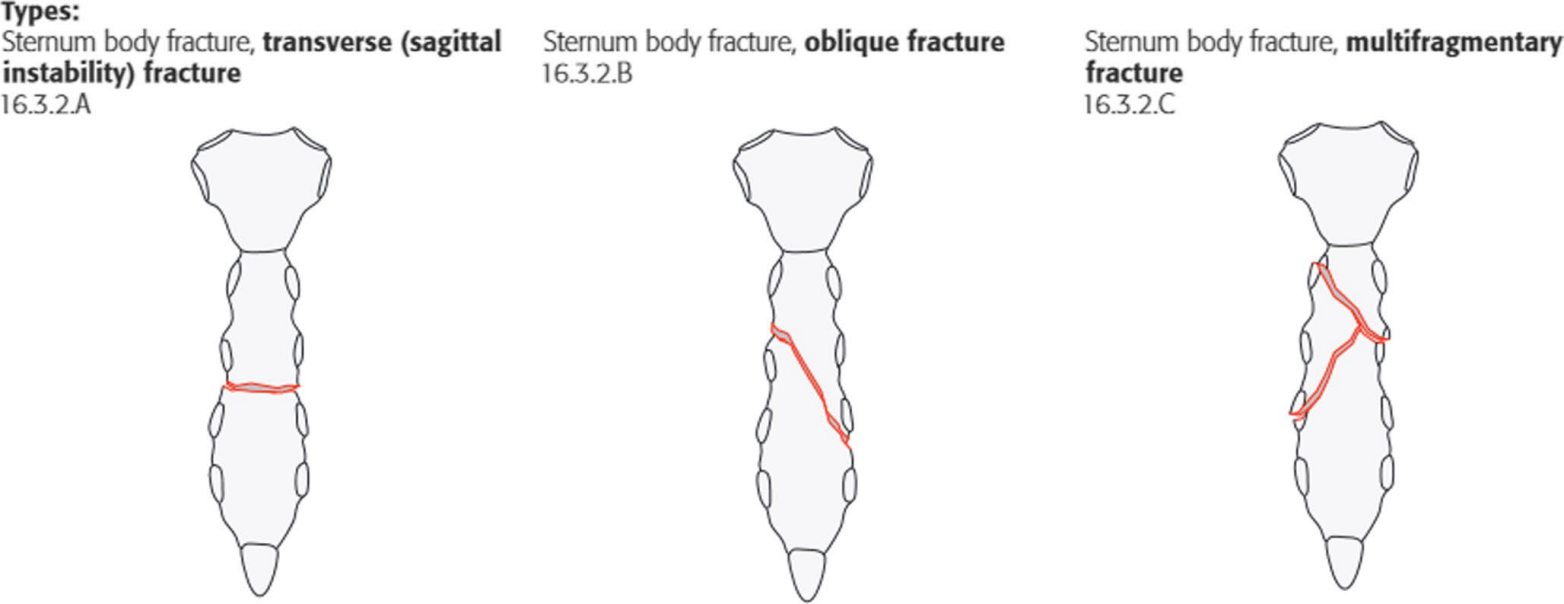
Fracture types at the corpus [17]

**Fig. 4:**
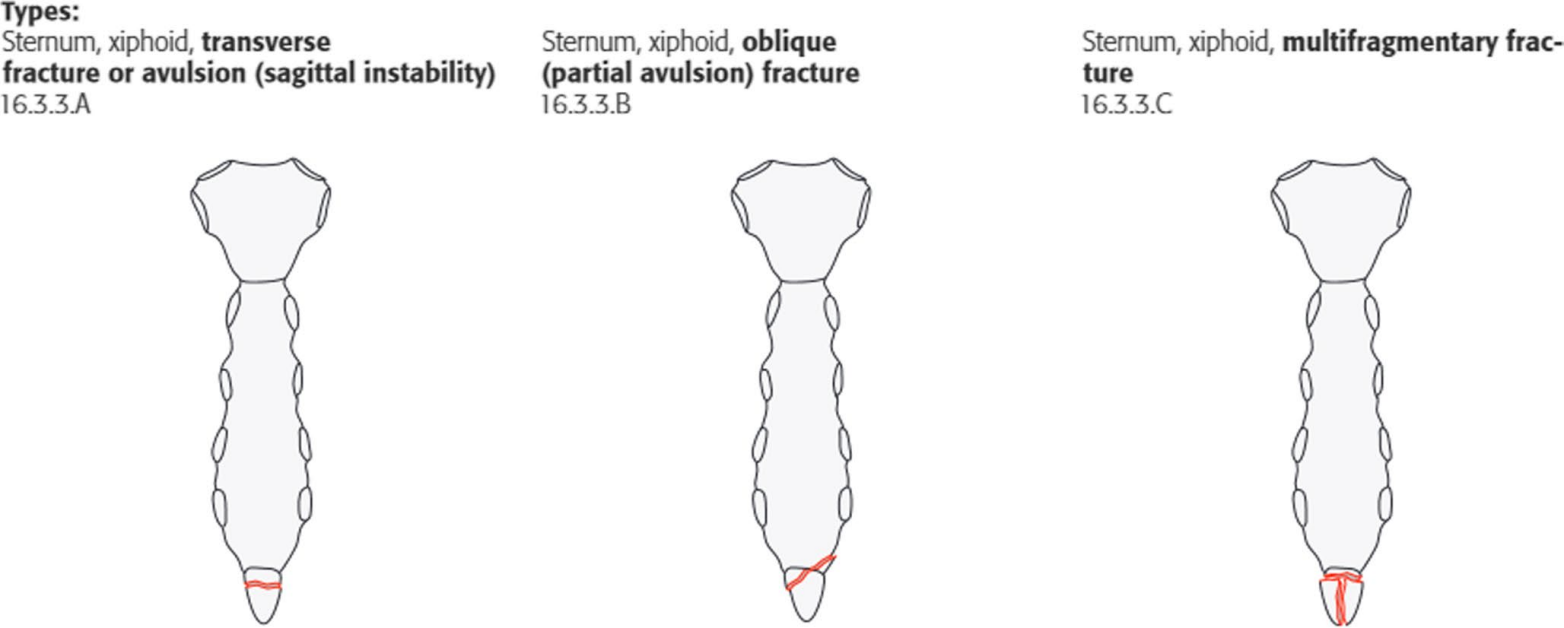
Fracture types at the xiphoid process [17]

For each fracture, the fracture was first classified as A, B or C according to the AO/OTA classification to be validated here (which was preliminary at the time of the study) (Figs. 2, 3, and 4).

**Fig. 5:**
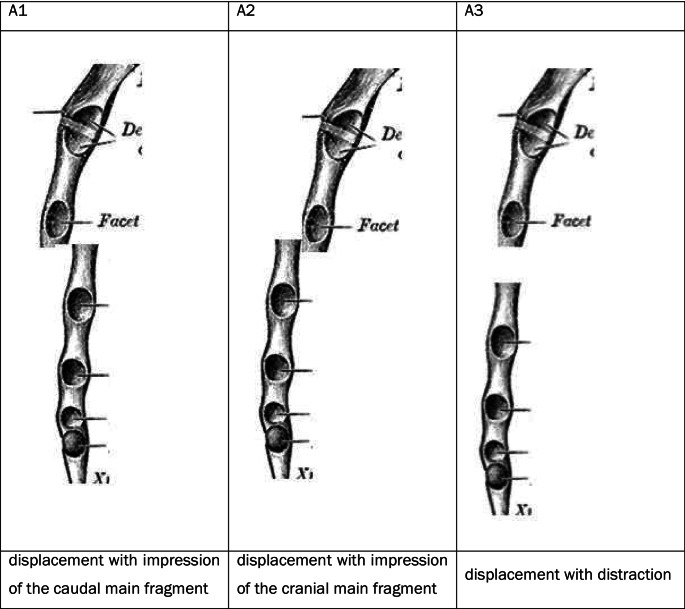
Subgroups in A-fractures of the corpus

**Fig. 6:**
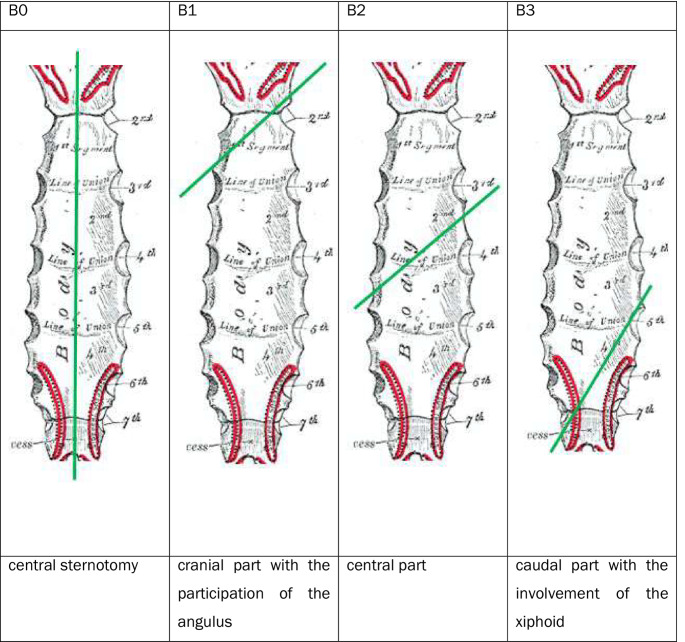
Subgroups in B-fractures of the corpus

**Fig. 7:**
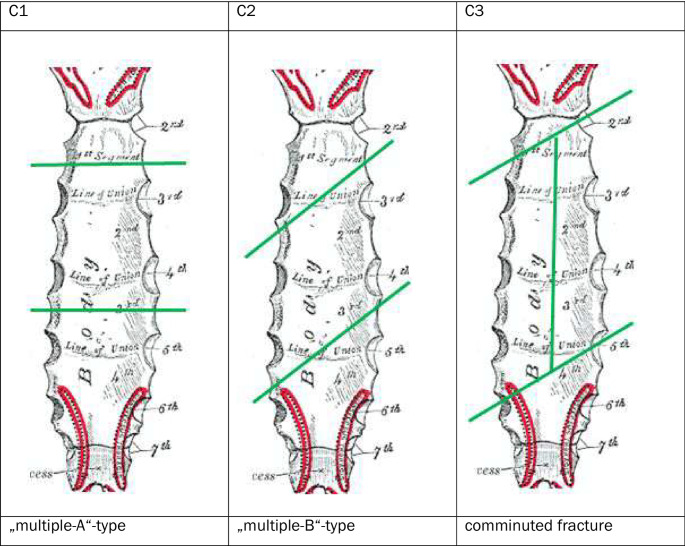
Subgroups in C-fractures of the corpus

The current AO classification does not yet define any subtypes or subgroups of A, B or C fractures. Subgroups of fractures were defined within the working group based on theoretical considerations, as shown in the diagram below. As part of the classification into A, B or C fractures, additional attention was paid to the proposed types and the types identified in the course of the work (Figs. 5, 6, and 7).

Initially, no subgroups were defined for the region of the manubrium or the xiphoid process, in the course of the analysis, the fractures of the manubrium sterni and those of the Proc. xiphoideus were also examined for the proposed subtypes.

The viewer program Synedra View (synedra information technologies GmbH, Innsbruck). was used to evaluate and measure the CT data.

The statistical analysis was carried out using Microsoft Excel for Office 365 (Microsoft Office 2021, version 16, Microsoft Corp., Redmond, Washington) and IBM SPSS Statistics 25 (IBM Inc., Armonk, NY, USA).

## Results

**Fig. 8:**
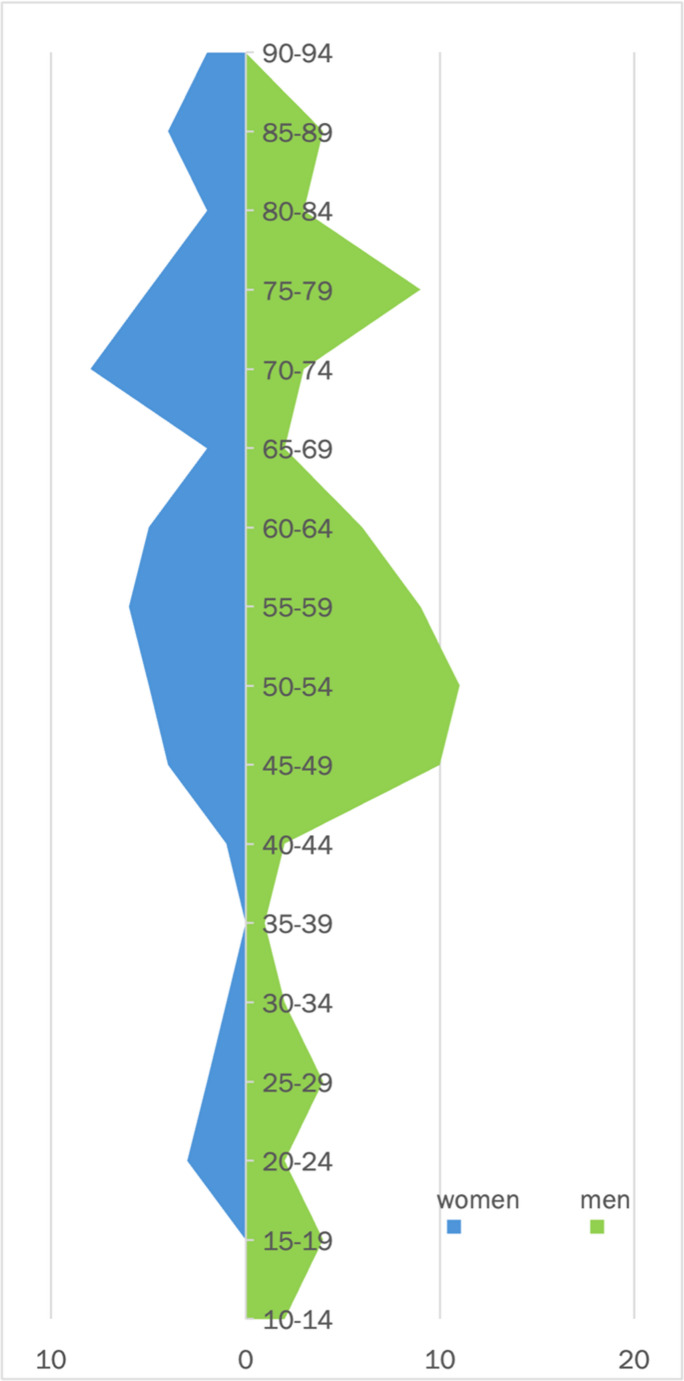
Age pyramid of the collective

The 124 patients of the entire anterior collective were divided into 60% (74) men and 40% (50) women. The mean age was 57 years [10;94; ±20]. The age distribution is shown in the graphs below. In the young men, there was a double peak with a peak at the age of majority and around the age of 30 (Fig. 8).

Of the 116 analyzed patients, 30.2% (35) had an isolated fracture of the sternal bone without additional fracturs of the thorax. 63.8% (74) showed additional fractures in the lateral sector (38 with bilateral fractures, 36 with unilateral fractures, of which 21 on the right and 15 on the left hemithorax). In 19% (22) additional fractures were detected in the posterior area. 24.1% (28) had additional fractures of the rib cartilage (9 bilateral, 19 unilateral, of which 11 on the right hemithorax and 8 on the left hemithorax). 38.8% (45) patients had additional fractures in only one sector (3 on the rib cartilage, 41 in the lateral segment and two in the posterior segment), 25.0% (29) had fractures in two additional sectors (16 anterior and lateral, 1 anterior and posterior, 12 lateral and posterior). 6.0% (7) had fractures in all three segments.

**Fig. 9:**
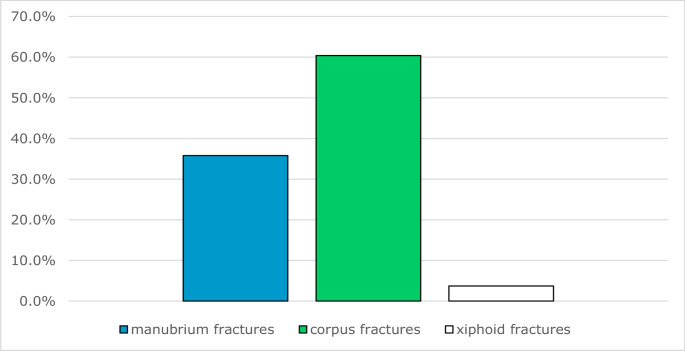
Distribution of sternal fractures to the individual sternum parts

The 116 patients with sternal fractures showed a total of 134 individual fractures. These were divided into 35.8% (48) manubrium fractures, 60.4% (81) fractures of the corpus sterni and 3.7% (5) fractures of the xiphoid process. 14.7% (17) of patients with sternal fractures had fractures in more than one part of the sternum. One patient had fractures in all three parts of the sternum, and 87.5% (14) of the remaining 16 patients had a combination of manubrium and corpus fracture. 12.5% (2) offered a combination of fracture of the corpus and proc. xiphoideus. A combination of fracture of manubrium and xiphoid process could not be found (Fig. 9).

The 48 manubrium fractures were classified into AO classes A, B and C as described above. There were 41.7% (20) A fractures, 43.8% (21) B fractures and 14.6% (7) C fractures.

Overall, 37.5% (18) of manubrium fractures were dislocated, including 40% (8) of A fractures, 20% (4) of B fractures, and 85.7% (6) of C fractures.

The 81 corpus fractures could be divided into 56.8% (46) A fractures, 29.6% (24) B fractures and 13.6% (11) C fractures.

51.9% (42) were displaced, including 58.7% (27) of A fractures, 29.2% (7) of B fractures, and 72.7% (8) of C fractures.

**Fig. 10:**
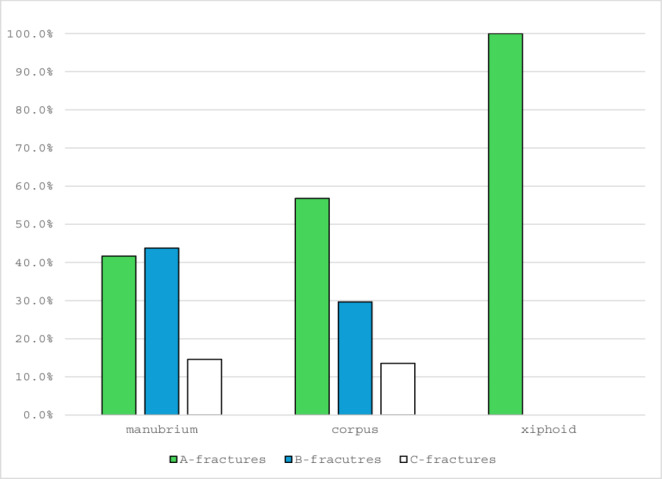
Distribution of sternum fractures per sternal part

**Fig. 11:**
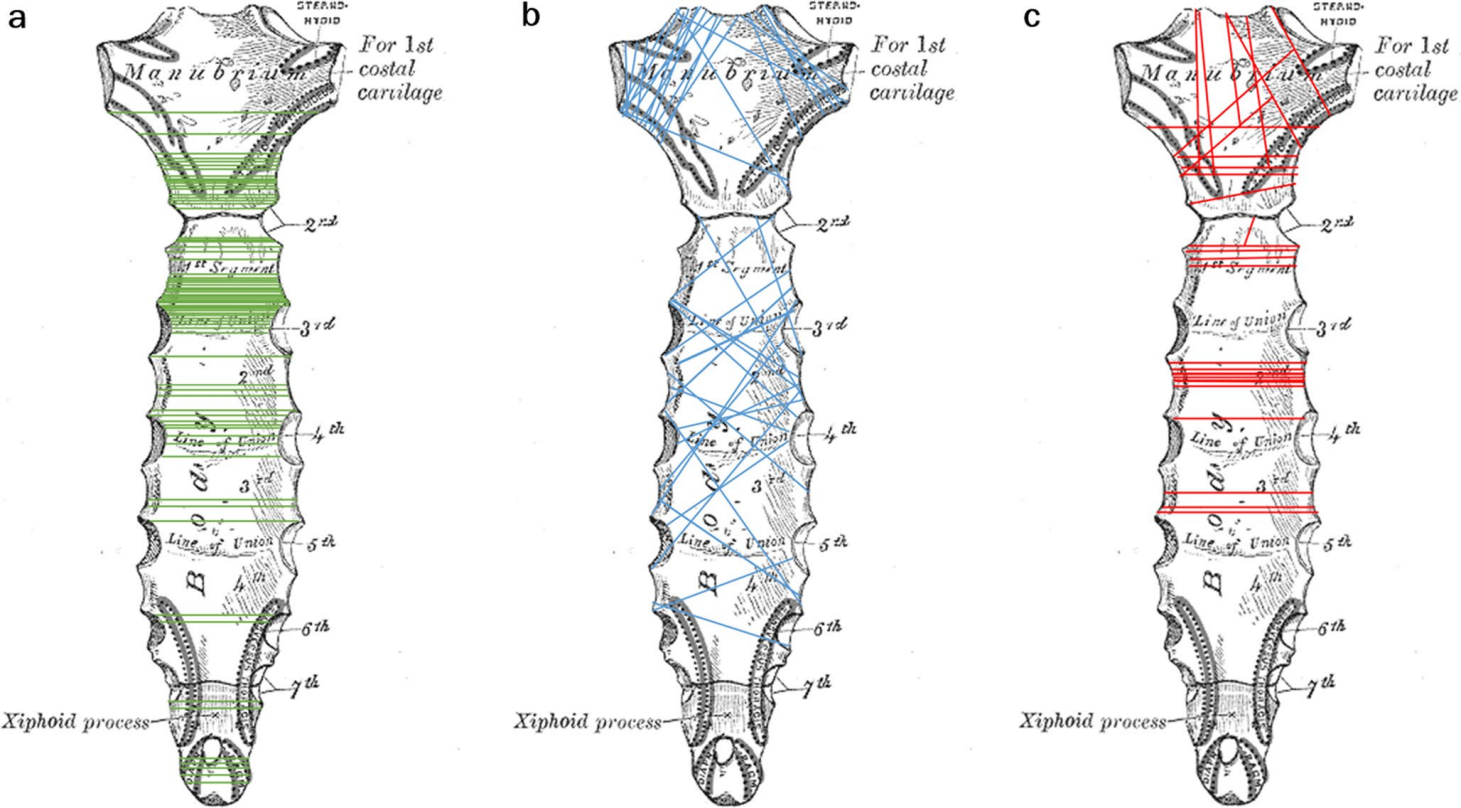
Distribution of the fractures over the sternal bone; **a** type A fractures; **b** type B fractures, **c** type C fractures

The five xiphoid fractures were 100% A-fractures, a B- or C-fracture did not occur in the present collective. 60% (3) showed a dislocation (Figs. 10 and 11).

The distribution of the fractures in relation to the anatomical location was as follows:

In the area of the manubrium sterni, 100% of the type A fractures were localized in the 1 st intercostal space.

Of the B fractures of the manubrium sterni, 95% involved two intercostal spaces, 5% of the fractures involved only one intercostal space.

The distribution of type A fractures in the area of the corpus sterni was as follows: 47.8% in the 2nd intercostal space, 19.6% in the 3rd intercostal space, 19.6% in the 4th intercostal space, 8.7% in the 5th intercostal space and 4.3% in the 6th intercostal space.

Of the B fractures of the corpus sterni, 16.7% involved one intercostal space, 66.7% involved two intercostal spaces and 16.7% involved three intercostal spaces. A fracture extending over more than three intercostal spaces was not found.

60% of the B fractures that extended over 2 ICRs ran from the 2nd to the 3rd ICR, 20% from the 3rd to the 4th ICR and 10% each from the 4th ICR to the 5th ICR and from the 5th to the 6th ICR.

The B fractures, which extended over 3 ICRs, ran 50% from the 3rd to the 5th ICR and 50% from the 2nd to the 4th ICR.

All type A fractures of the Proc. xiphoideus were localized in the 7th intercostal space.

The subgroups were first divided into the proposed types. As mentioned above, considerations were made during the work on further sub-groups, which are briefly listed here and will be examined in more detail in the subsequent discussion.

**Fig. 12:**
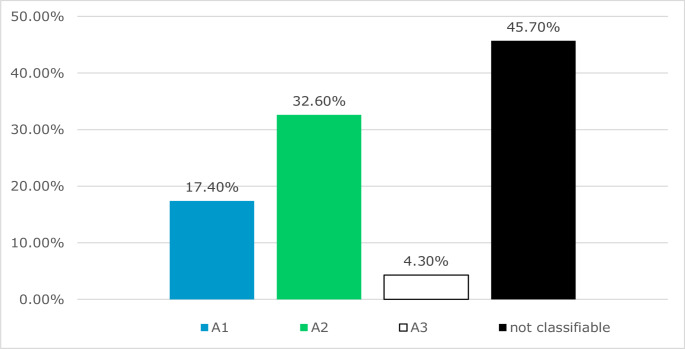
Analysis of the proposed subgroups in A-fractures of the corpus sterni

The 46 A-fractures of the corpus sterni could be classified into 15.2% (8) A1 fractures, 30.4% (15) A2 fractures and 2.2% (2) A3 fractures. 45.7% (21) fractures could not be assigned to any type in the absence of dislocation (Fig. 12).

**Fig. 13:**
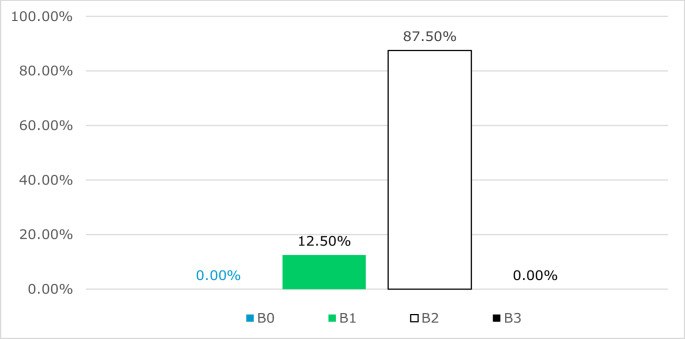
Analysis of the proposed subtypes in B fractures of the corpus sterni

The 24 B fractures of the corpus could be divided into 12.5% (3) B1 fractures and 87.5% (21) B2 fractures. A B0 and a B3 fracture were not found in the present collective. The course of the 21 B2 fractures was also examined. 66.7% (14) were caudal from the right cranial to the left, and 33.3% (7) were caudal from the left cranial to the right (Fig. 13).

**Fig. 14:**
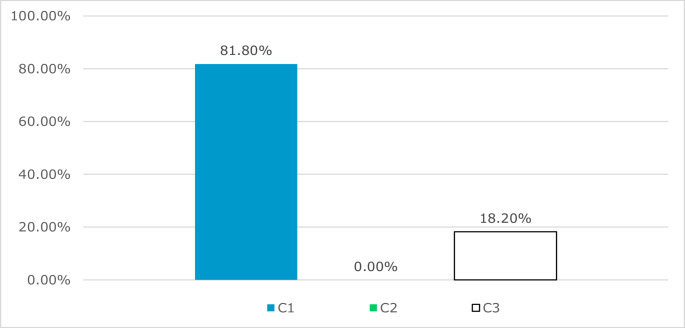
Analysis of the proposed subgroups in C fractures of the corpus sterni

Of the 11 C fractures, 81.8% (9) were C1 fractures, 0% (0) were C2 fractures and 18.2% (2) were C3 fractures (Fig. 14).

Initially, no subclassification was proposed in the area of the manubrium. With regard to belt trauma, the B-fractures were analyzed in more detail. In 20 B-fractures, 45% (9) were found with a fracture from cranial left to caudal right and 55% (11) with fracture progression from cranial right to caudal left.

The low number of fractures of the xiphoid process as well as only one existing entity (A-fractures, see above) did not allow for a meaningful subclassification.

## Discussion

The age structure corresponds to the age pyramid from the years of the analysis (2010–2016). Thus, the patient collective in this study is representative in its structure for polytraumatized patients in general [19] and for patients with sternal fractures [20–22] in comparison. This allows the following conclusions about thoracic trauma and fractures of the anterior chest wall beyond the present analyzed collective.

The above data have shown that sternal fractures rarely occur as an isolated fracture and in most cases are associated with concomitant injuries to the lateral chest wall. There are only a few case reports of isolated fractures of the sternum in the literature. In patients with clinical and radiological suspicion of an injury to the anterior thoracic wall, special attention must be paid to the entire thorax. Although sternal fractures are a rare entity overall, accounting for 3–8% of trauma patients [23], a vice-versa correlation between fractures of the posterior or lateral chest wall and fractures of the anterior sector should not be ruled out. A detailed analysis of this question is the subject of a further study by the working group [24].

The above results show that the general classification into A, B and C fractures in the manubrium and corpus is both clinically relevant and conclusive about fracture severity. This could not be confirmed for the xiphoid due to the small number of cases. There is still room for improvement in subtyping. The subdivision of A-fractures according to the type of dislocation seems to make sense, as theoretical considerations can be used to draw conclusions about the underlying trauma mechanism and possible concomitant injuries. For example, an A1 fracture suggests a direct impact on the caudal part of the respective stellar part, while an A2 fracture suggests an impact on the cranial part. Distraction fractures in the sense of the A3 fracture described above suggest a hyperextension mechanism, which can be accompanied by an injury to the large abdominal and thoracic vessels as well as an additional injury to the spine.

However, it could be found that some entities could not be classified or could only be classified to a limited extent. Since the dislocation in CT is often only a snapshot and is often dependent on positioning and breathing, especially in the thorax area, it is not possible to draw conclusions about the mechanism in all cases. In this case, it is important to have detailed knowledge of the accident event through one’s own or third-party anamnesis in order not to overlook accompanying injuries.

The absence of A-fractures of the manubrium sterni above the 1 st rib and the accumulation of fractures in the lower third of this part of the sternum could be explained by the anatomical structure and the varying thickness of the manubrium at different heights. The thickest region is located at the level of the lower clavicle joint surfaces, the thinnest in the transition from the upper two thirds to the lower third of the manubrium [25], which correlates with the location of the A-fractures and identifies the thinner areas of the manubrium as weak points.

In the area of the B fractures, only few fractures involving the angulus and no fracture of the transition to the xiphoid could be found. In theory, these fractures are clinically relevant. In particular, the location of these fractures plays a role in the choice of osteosynthesis material. While B2 fractures can usually be stabilized with a long standard plate due to their location, T-plates or anatomical plates are often required for stabilization of B1 and B3 fractures. The occurrence of B1 and B3 fractures should therefore be examined across centers and in a larger collective.

B0 fractures could not be shown in the present collective, and there is also no description of this entity in the literature in the context of trauma-associated fractures. Since these occur mainly iatrogenically in the context of sternotomies, this type has no relevance in the context of fracture typing and should therefore not be part of the classification.

**Fig. 15:**
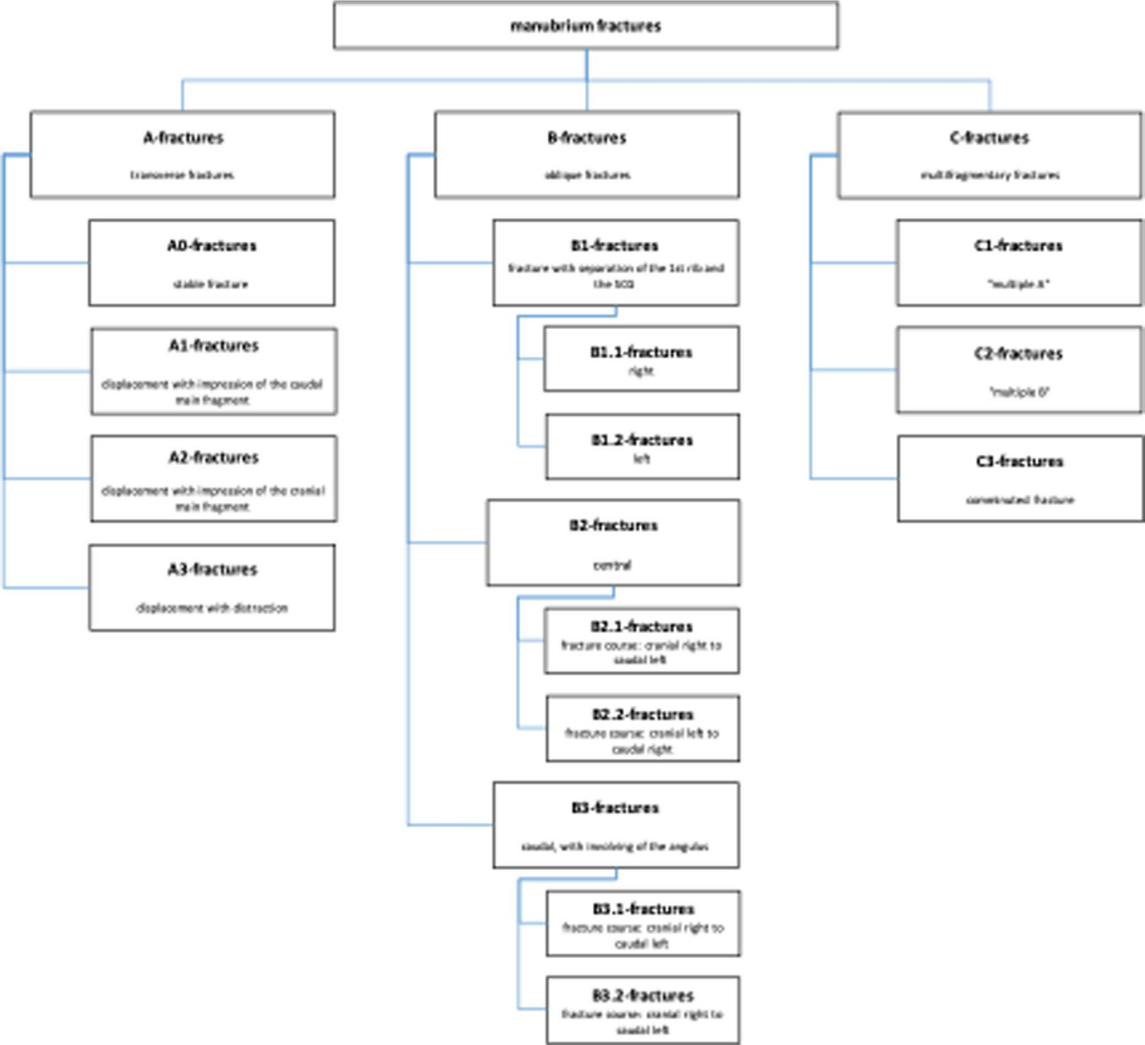
Alternative proposal for the classification of fractures of the manubrium

**Fig. 16:**
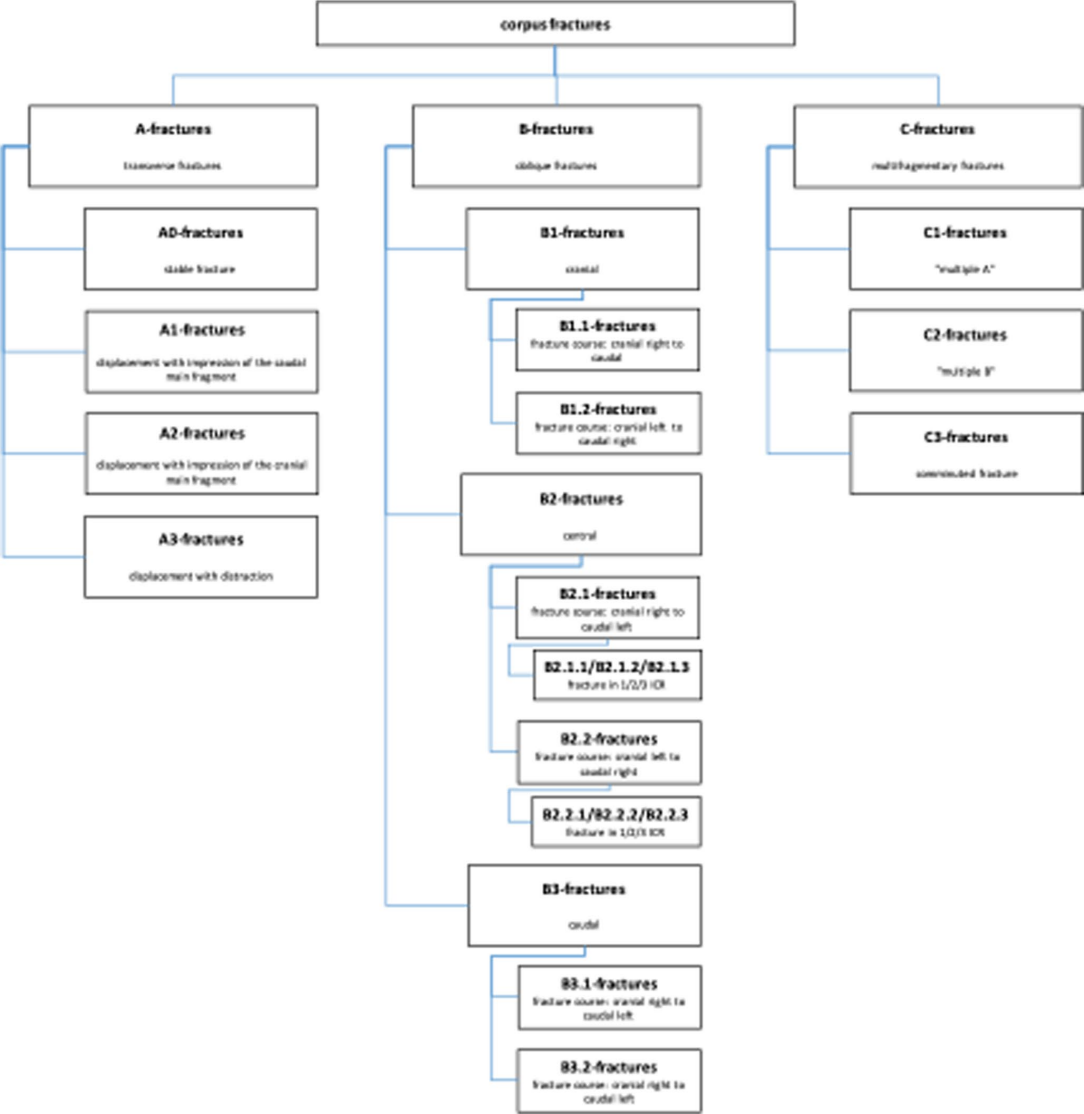
Alternative proposal for the classification of fractures of the corpus

B-fractures in the manubrium and corpus are often associated with belt injuries in the context of car accidents. The course of the fracture usually corresponds to the course of the belt. In the driver’s case, the fracture (assuming left-hand drive) runs from cranial left to caudal right. In the case of the co-driver, the course is vice versa. From the course of the fracture, it may be possible to draw conclusions about the position in the car and the accident mechanism. This trend should be considered in the classification. Multi-fragment C-fractures are dislocated in most cases, as shown above. Instability can also be assumed in the case of slightly dislocated or non-dislocated C-fractures. A division into dislocated and undislocated therefore seems to make little sense from a clinical point of view and for the sake of clarity. These considerations apply equally to the manubrium and the corpus. Due to the lack of data and the assumed low clinical relevance of fractures in this area, the xiphoid is simply divided into A, B and C. An overview of the considerations and a proposal for classification is shown in the following graphics (Figs. 15 and 16).

## Treatment options

Sternal fractures are rare injuries, and current literature offers limited evidence—particularly regarding long-term outcomes. Nonetheless, therapeutic strategies, both conservative and operative, have been established based on clinical experience and biomechanical principles.

Isolated, non-displaced sternal fractures are most managed non-operatively. Conservative treatment consists of adequate pain control, respiratory physiotherapy to maintain pulmonary function and activity modification. The aim is to avoid complications such as atelectasis or pneumonia while allowing natural bone healing. Provided that no displacement or mechanical instability is present, outcomes with conservative treatment are generally favorable. Harston and Roberts report satisfactory results in the majority of patients managed non-surgically, emphasizing the importance of functional respiratory management and analgesia [26]. However, in displaced fractures or fractures subject to persistent mechanical stress due to respiratory movements, conservative therapy may be insufficient. These cases are associated with delayed union or progression to pseudarthrosis. Poor healing in patients with significant sternal displacement or instability when treated conservatively [27]. Non-displaced fractures need follow up examinations with clinical examination (regarding instability, hunched back deformity, restricted movement due to pain) and lateral x-ray in sitting position.

Surgical stabilization of sternal fractures is reserved for cases where conservative therapy fails or where specific anatomical and clinical indications are present [26, 28]. These include:

- Persistent sternal deformity after trauma.
- Non-union or pseudoarthrosis after more than six weeks of conservative management.
- Chronic or severe anterior chest wall pain lasting two to eight weeks.
- Displaced fractures (especially overlapping or malrotated fragments).
- Mechanical instability of the anterior chest wall.
- Sternal fractures associated with flail chest or multiple rib fractures.
- Functional limitations due to pain or instability during respiration or movement.

Mayberry et al. defined early surgical stabilization indications as persistent deformity, non-union, and disabling pain unresponsive to conservative measures [28].

Surgical treatment has been associated with improved stability, faster pain reduction, and a lower risk of non-union compared to conservative management in appropriately selected patients [26].

To date, the earliest known report of surgical fixation of a sternal fracture was published by McKim in 1943, who used a Kirschner wire for stabilization [29]. In the subsequent decades, various techniques have been explored, including intramedullary fixation, external fixation devices, wire cerclage, and traditional plate osteosynthesis. More recently, most published studies focus on the use of locked plating systems, particularly low-profile titanium plates that enable secure fixation through depth-limited drilling [30]. Earlier investigations have demonstrated that compared to wire-based techniques and other forms of osteosynthesis, sternal plating offers superior biomechanical stability, enhances chest wall function, reduces the risk of non-union, and promotes more effective bone healing [31, 32]. A systematic review found that in 83% of surgical cases, plate osteosynthesis was used [33]. Retrospective analyses suggest that the use of double 3.5 mm titanium locking compression plates yields excellent long-term outcomes and minimal complication rates—even in polytraumatized patients [34].

Therapeutic approaches for sternal fractures are based on case series and biomechanical principles. Plates with locking screws have proven to be the method of choice for osteosynthesis of sternal fractures The standard surgical approach is via a longitudinal anterior midline incision tailored to the fracture location. Subperiosteal exposure allows for anatomical reduction of fragments. Depth-limited drilling is used to prevent injury to retrosternal structures. Fixation is performed with titanium locking plates, which provide multidirectional stability and reduce micromotion during respiration [31].

**Fig. 17:**
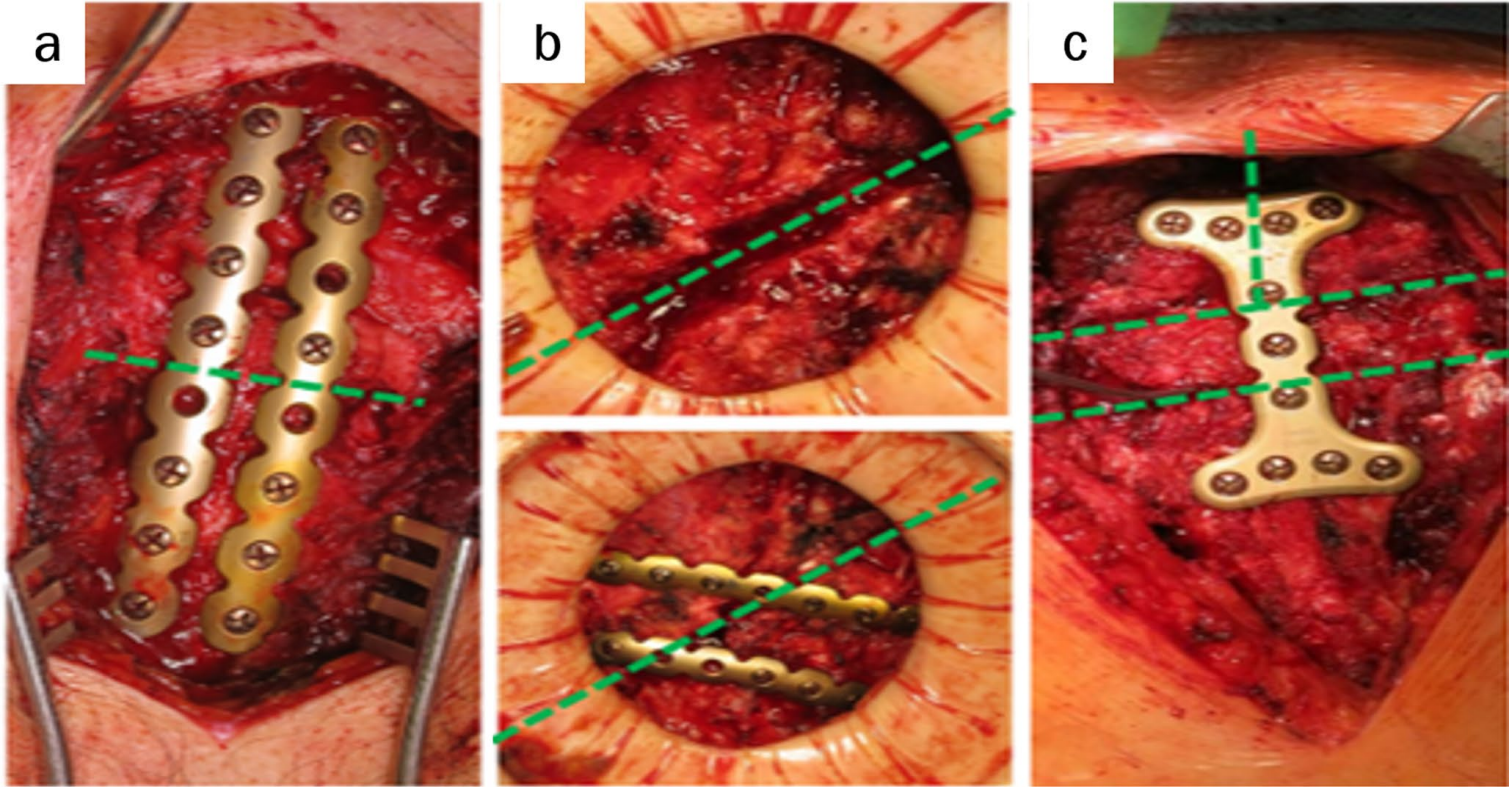
Plating of fractures of the manubrium, **a** longitudinal double-plating of an A1-fracture; **b** transversal plating of a B1-fracture; **c** H-shaped plating to address multidirectional instability of a C-fracture

**Fig. 18:**
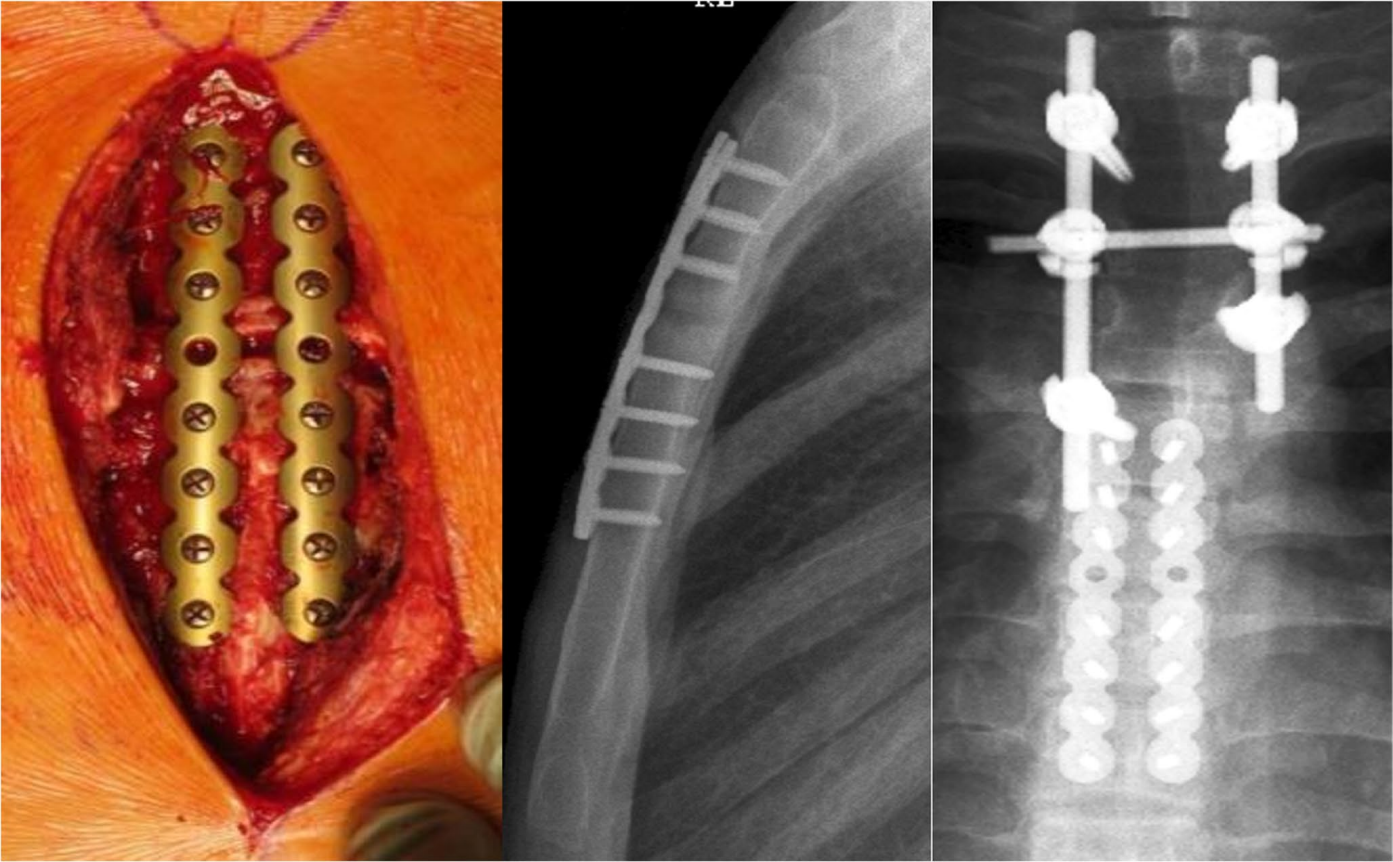
Double plating of an A2-fracture of the corpus

**Fig. 19:**
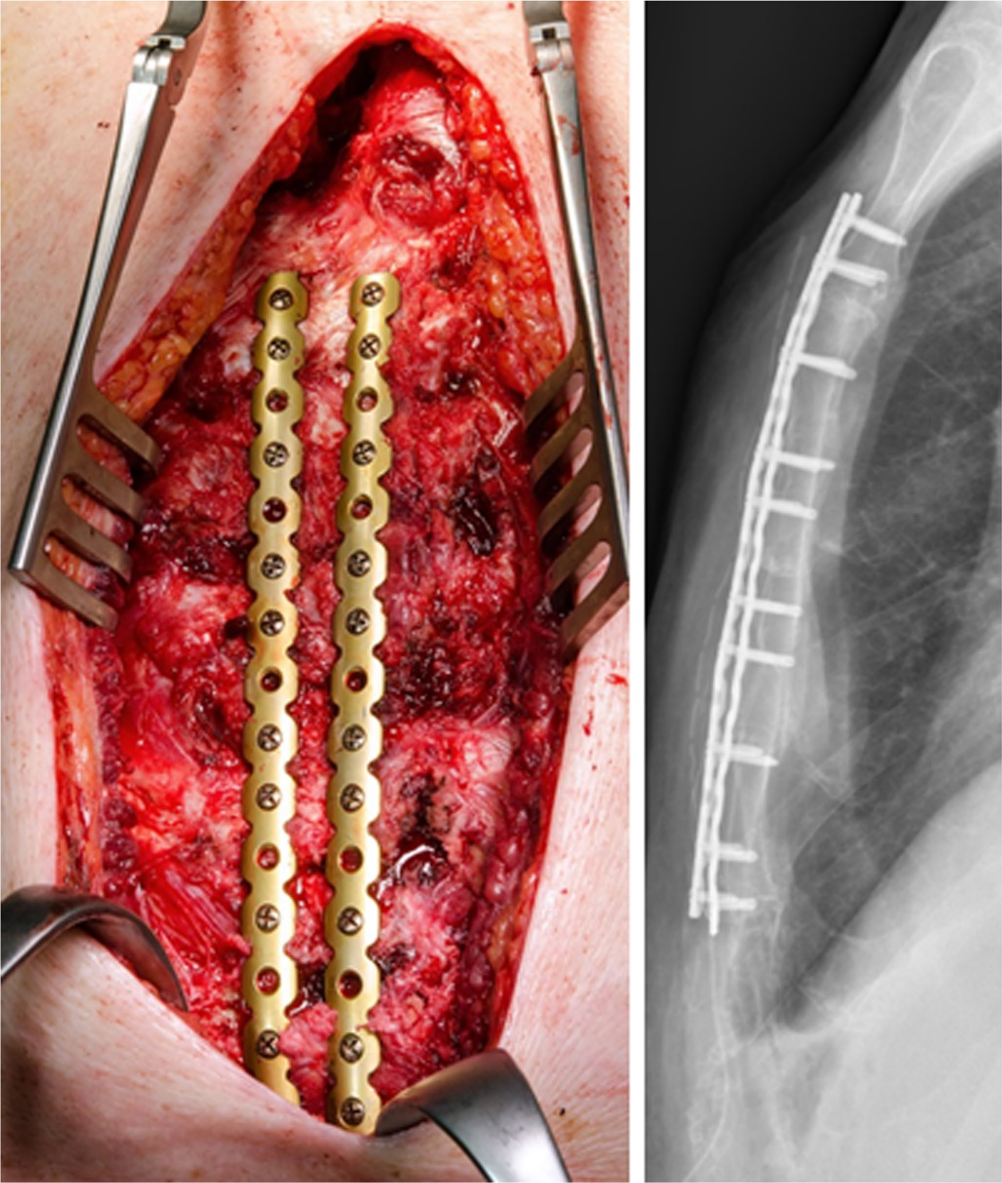
C-fracture of the corpus with longitudinal double-plating extending to the manubrium

Fixation strategies depend on the type and location of the fracture [32]. Transverse or oblique fractures of the sternal corpus are typically stabilized with longitudinal plates. Oblique manubrial fractures or sternocostal separations are treated with transverse plates that bridge the rib–sternum–rib axis, restoring the continuity of the anterior chest wall. Complex fractures may require longer plates or custom-shaped constructs such as T- or H-shaped plates to address multidirectional instability (Figs. 17, 18, and 19).

The anatomical location of the fracture influences surgical planning [35]:

- Upper manubrium (jugular notch to first rib): Transverse rib-to-manubrium plating to address anterior chest wall instability.
- Central manubrium: Often requires bilateral longitudinal plates for broad stabilization.
- Lower sternum: Short rib insertion angles complicate fixation; costosternal plating or T-/I-shaped plates may be necessary for adequate anchorage (Tables 2 and 3).

**Table 2:** Treatment recommendations for the proposed fracture classification of the manubrium

Manubrium fractures	
Fracture type	Treatment
A-fractures	
A0-fractures	conservative treatment, follow up examination (lateral x-ray in sitting position)
A1-fractures	longitudinal (double) plating
A2-fractures	longitudinal (double) plating
A3-fractures	longitudinal (double) plating
B-fractures	
B1-fractures	transverse plates bridging the rib–sternum–rib axis
B2-fractures	transverse plates bridging the rib–sternum–rib axis or longitudinal (double) plating
B3-fractures	transverse plates bridging the rib–sternum–rib axis or longitudinal (double) plating
C-fractures	
C1-fractures	longitudinal (double) plating
C2-fractures	transverse plates bridging the rib–sternum–rib axis, T- or H-shaped plates to address multidirectional instability
C3-fractures	T- or H-shaped plates to address multidirectional instability

**Table 3:** Treatment recommendations for the proposed fracture classification of the corpus

Corpus fractures	
Fracture type	Treatment
A-fractures	
A0-fractures	conservative treatment, follow up examination (lateral x-ray in sitting position)
A1-fractures	longitudinal (double) plating
A2-fractures	longitudinal (double) plating
A3-fractures	longitudinal (double) plating
B-fractures	
B1-fractures	transverse plates bridging the rib–sternum–rib axis or longitudinal plates (T-shaped)
B2-fractures	transverse plates bridging the rib–sternum–rib axis or longitudinal (double) plating
B3-fractures	transverse plates bridging the rib–sternum–rib axis or T-shaped plates
C-fractures	
C1-fractures	longitudinal (double) plating (consider from Manubrium to xiphoid)
C2-fractures	C1 or (multiple) transverse plates bridging the rib–sternum–rib axis, or combination of both directions
C3-fractures	C2 and additional combination of special plates (T-Plates, H-Plates, Ladder-plates etc.), to address multidirectional instability; consider lateral extensions to the adjacent ribs

## Limitation

The data collected were obtained from a single center. Further multicenter studies are necessary to support the above hypotheses and to validate the proposed classification below. Additionally, in this analysis, the progression of the fracture in the sagittal plane across potential subgroups was not captured. A-fractures, particularly those of the sternal body, can also extend across intercostal spaces. Further research is necessary to determine how to classify those types correctly.

Moreover, the treatment recommendations provided are based on a review of the literature that does not always specifically address the individual fracture types described above. Therefore, further biomechanical studies are needed to validate and refine these therapeutic suggestions.

## Conclusion

Sternal fractures, though relatively uncommon, present a complex clinical challenge due to their frequent association with additional thoracic injuries and varied fracture morphologies. The proposed classification provides a structured framework that reflects clinical relevance and fracture severity. Conservative treatment remains effective for non-displaced fractures, but surgical stabilization is essential in cases of displacement, instability, or failed conservative therapy. Locking plate osteosynthesis has emerged as the gold standard, offering superior biomechanical stability and favorable outcomes. Further multicenter and biomechanical studies are necessary to refine treatment algorithms and validate classification-based therapeutic recommendations.

## Data Availability

The data that support the findings of this study are available from the corresponding author upon reasonable request.
